# Lung Ultrasound-Guided Non-Fiberobronchoscopic Bronchoalveolar Lavage for Neonatal Atelectatic Pulmonary Disease Treatment: Clinical Practice Guidelines Based on International Expert Consensus

**DOI:** 10.3390/diagnostics16172712

**Published:** 2026-08-25

**Authors:** Jing Liu, Ya-Li Guo, Peng Jiang, Xian Zhang, Bi-Ying Deng, Zun-Jie Liu, Xiao-Xiao Wang, Yuan Hong, Xiao-Ling Ren, Meng-Ru Zhao, Ning Li, Cai-Xuan Xie, Qiong Meng, Chu-Ming You, Zhen-Yu Liang, Rui-Yan Shan, Jia-Gen Cen, Shuo Li, Wen-Ping Wang, Li-Li Zang, Ying-Jun Wang, Lu Liu, Wei Fu, Yi-Na Ye, Xiao-Xia Li, Ling-Yun Bao, Zai-Li Feng, Ayinuer Maimaitili, Erich Sorantin, Kai-Sheng Hsieh, Dalibor Kurepa, Jovan Lovrenski, Piotr Kruczek, Stefano Nobile, Tsu F. Yeh, Giovanni Volpicelli, Pradeep Suryawanshi, Abhay Lodha, Yogen Singh, on behalf of the Paediatric Medicine Branch of Asia–Pacific Health Association, the Neonatal Critical Care Medicine Branch of Beijing Association of Holistic Integrative Medicine, the Lung Ultrasound Technology Extension Expert Group of China National Health Association

**Affiliations:** 1 Department of Neonatology and NICU, Beijing Obstetrics and Gynecology Hospital, Capital Medical University, Beijing Maternal and Child Health Care Hospital, Beijing 100026, China; 2 Department of Pediatrics, Second Affiliated Hospital of Kunming Medical University, Kunming 650034, China; 3 Department of Neonatology, Dongguan Children’s Hospital Affiliated to Guangdong Medical University, Dongguan 523000, China; 4 Department of Pediatrics, The Affiliated Guangdong Second Provincial General Hospital of Jinan University, Guangzhou 510220, China; 5 Department of Neonatology, Yantai Yuhuangding Hospital, Yantai 264000, China; 6 Department of Neonatology, Qianxinan Prefecture People’s Hospital, Xingyi 562400, China; 7 Department of Ultrasound Medicine, Beijing Chao-Yang Hospital, Capital Medical University, Beijing 100020, China; 8 Department of Neonatology, Beijing Chao-Yang Hospital, Capital Medical University, Beijing 100020, China; 9 Department of Neonatal Intensive Care Unit, Zhoukou Central Hospital, Zhoukou 466000, China; 10 Department of Neonatology and NICU, Beijing Chao-Yang District Maternal and Child Health Care Hospital, Beijing 100021, China; 11 Department of Pediatrics, The Seventh Affiliated Hospital, Southern Medical University, Foshan 528244, China; 12 Department of Ultrasound, Liaocheng Dongchangfu District Maternal and Child Health Hospital, Liaocheng 252000, China; 13 Department of Neonatology, Kunming Children’s Hospital, Kunming 650034, China; 14 Neonatology Department, The Affiliated Dehong Hospital of Kunming Medical University (The People’s Hospital of Dehong Prefecture), Mangshi 678400, China; 15 Department of Ultrasonography, Kizilsu Kergez Prefecture People’s Hospital, Atushi 854350, China; 16 Division of Pediatric Radiology, Department of Radiology, Medical University Graz, 8010 Graz, Austria; 17 Ultrasound Center, Children’s Hospital, China Medical University, Taichung 404328, Taiwan; 18 Division of Neonatology and Pulmonary Biology, Cincinnati Children’s Hospital and Medical Center, Cincinnati, OH 45229, USA; 19 Radiology Department, Faculty of Medicine Novi Sad, University of Novi Sad, 21102 Novi Sad, Serbia; 20 Department of Neonatology, Ujastek Medical Center, ul. Ujastek 3, 31-752 Cracow, Poland; 21 Neonatal Unit, Department of Mother, Child and Public Health, Fondazione Policlinico Universitario a. Gemelli IRCCS, Largo F. Vito 1, 00168 Rome, Italy; 22 Division of Neonatology and NICU, Cook County Children’s Hospital, University of Illinois, Champaign, IL 61801, USA; 23 Department of Medical and Surgical Science, Magna Græcia University, 88100 Catanzaro, Italy; 24 Department of Neonatology, Bharati Vidyapeeth University Medical College, Pune 411043, India; 25 The Section of Newborn Critical Care, Department of Pediatrics, Cumming School of Medicine, Royal College of Physicians of Edinburgh, University of Calgary, Edinburgh EH2 1JQ, UK; 26 Division of Neonatology, Department of Pediatrics, UC Davis Children’s Hospital, University of California, Sacramento, CA 95718, USA

**Keywords:** atelectatic pulmonary diseases, atelectasis, pneumonia, meconium aspiration syndrome, lung ultrasound, bronchoalveolar lavage, non-fiberobronchoscopic bronchoalveolar lavage, neonate, neonatal intensive care unit

## Abstract

Severe pulmonary diseases, including atelectasis, pneumonia, and meconium aspiration syndrome, are major causes of neonatal respiratory distress, weaning difficulties, ventilator or oxygen dependence, prolonged oxygen requirements, extended hospitalization, and poor prognosis. The lack of simple and effective treatment strategies seriously endangers the survival and health of newborns, particularly premature infants. Recent advances in lung ultrasound (LUS) technology have made LUS-guided non-fiberobronchoscopic bronchoalveolar lavage (NFB-BAL) a promising solution. This approach addresses the limitations of conventional BAL in neonates by enabling accurate diagnosis, precise lesion localization, and dynamic procedural monitoring, hence reducing complications. Based on international expert consensus, these guidelines were developed to improve knowledge and promote the application of this technology and enhance operational standardization to ensure its effectiveness and safety. These guidelines contain 17 recommendations on 13 key clinical issues for reference and implementation in clinical practice. The wide application of these guidelines is expected to help significantly improve the prognosis of critically ill newborns with atelectatic pulmonary diseases.

## 1. Introduction

Atelectatic pulmonary diseases (APDs) are severe lung disorders characterized by extensive lung consolidation on ultrasound imaging. These diseases, which prevent the lungs from expanding normally, cause lung collapse and loss of normal function. In newborns, these diseases include severe atelectasis, severe pneumonia, and severe meconium aspiration syndrome (MAS) [1,2]. Due to different pathophysiological factors, APDs or atelectasis may be associated with severe respiratory difficulties, the need for ventilator support, difficulty in weaning from ventilator support, repeated intubation, ventilator dependence, oxygen dependence, a prolonged disease course, and poor prognoses in newborns and premature infants. Due to the lack of simple and effective treatment solutions, APDs endanger the lives and health of newborns and premature infants. Because of their immature pulmonary and cardiovascular systems, premature neonates are highly vulnerable during airway manipulation, even during brief procedures performed by experts. Bronchoscopy technology has been widely applied in adult, child, and newborn treatment, and the use of bronchoscopic techniques to obtain bronchoalveolar lavage (BAL) specimens from an affected lung area with severe lesion is a superior strategy to that based exclusively on clinical evaluation [3,4,5,6,7,8,9]. Despite its efficacy and relative safety in patients, bronchoscopy has yet to become a preferred option for respiratory management in newborns because of its associated technical difficulties, high risk, and potential complications [10,11,12,13,14]. A systematic review and analysis involving 107,969 bronchoscopy procedures revealed that its complications include but are not limited to complications of local anesthesia (0.3–0.5%), hypoxemia (0.2–21%), arrhythmia (1.0–10.0%), post-biopsy bleeding (0.12–7.5%), pneumothorax or pneumomediastinum (1.0–6.0%), fever (0.9–2.5%), and even death (0.1–0.2%) [12], and the incidence of complications is further increased if bronchoscopic alveolar lavage is performed simultaneously [14]. Internationally, in clinical newborn and premature infant treatment, non-fiberobronchoscopic bronchoalveolar lavage (NFB-BAL) is preferred for cytological and microbiological pathogen diagnosis, as it is equally as effective as traditional bronchoscopic lavage [15,16,17]. In recent years, lung ultrasound (LUS)-guided NFB-BAL for neonatal APD has not only overcome the shortcomings of traditional BAL, but also achieved remarkable clinical results, significantly reducing or even avoiding the use of bronchoscopy [2,18,19,20,21]. To promote and standardize the clinical application of this new technology, international experts in this field have proposed clinical practice guidelines.

## 2. Methods and Procedures for Formulating the Guidelines

### 2.1. The Initiating and Organizing Units of the Guidelines

These guidelines were jointly organized and initiated by the Pediatric Medicine Branch of the Asia–Pacific Health Association, the Neonatal Critical Care Medicine Branch of the Beijing Association of Holistic Integrative Medicine, and the Lung Ultrasound Technology Extension Expert Group of the China National Health Association. The Department of Neonatology and NICU, Beijing Obstetrics and Gynecology Hospital, affiliated with Capital Medical University, was responsible for the entire guideline development process.

### 2.2. Guideline Registration

These guidelines were registered on the International Practice Guideline Registry and Transparency Platform (http://guidelines-registry.cn/) on 17 December 2024, with the registration number PREPARE-2024CN1189.

### 2.3. Guideline Formulation Method

These guidelines were developed based on the methods provided in the “WHO Handbook for Developing Guidelines (Second Edition)”, the “Appraisal of Guidelines for Research & Evaluation II” (AGREE II), and the “Reporting Items for Practice Guidelines in Healthcare” (RIPGH) [22,23].

### 2.4. Establishment of a Preparatory Group for Guideline Writing and Convening of a Preparatory Meeting

A preparatory group consisting of five experts was established, and three online preparatory meetings were held on 20 December 2024, 26 December 2024, and 5 January 2025. Required activities were delegated among the preparatory group members, with each member responsible for expert selection, literature searches, problem formulation and organization, and writing the initial draft. Key clinical issues that might have been involved when reaching consensus were screened and carefully discussed.

### 2.5. Selection of Experts to Formulate the Guidelines

A total of 39 experts participated in this work, including 32 members of the core panelists and 7 members of the extended advisory group. All of the experts met at least one of the following criteria: (1) proficiency in neonatal LUS techniques; (2) part of a department where NFB-BAL was performed under ultrasound guidance and clinical experience in this technology; (3) had published papers as a first author, corresponding author, or co-author in core Chinese journals or journals indexed in PubMed, Web of Science, etc.

### 2.6. Literature Search and Updates

The literature search team conducted the literature search and screening. Based on the population (newborns with APDs), intervention (NFB-BAL), comparison (no NFB-BAL), outcome (the results of NFB-BAL, side effects) (PICO) principle, after analyzing the key clinical issues to be addressed, a systematic search was conducted using a combined search strategy of MeSH terms and free words, which included lung ultrasound, chest ultrasound, point-of-care lung ultrasound, ultrasound monitoring, chest X-ray, lung disease, neonate, bronchoalveolar lavage, non-bronchoscopy, pneumonia, meconium aspiration, atelectasis, atelectatic pulmonary diseases, etc. The search platforms included PubMed, MEDLINE, Web of Science, the Cochrane Library, the Chinese Biomedical Literature Database, the China National Knowledge Infrastructure, the Wanfang Database, and the Chinese Medical Journal Full-text Database. Only Chinese and English literature was included. The literature search was performed every two months until two months before submission, and several new documents were supplemented during the revision process to ensure that the latest evidence was included.

### 2.7. Inclusion and Exclusion Criteria for the Literature: Quality Assessment and Evidence Extraction

Two members of the evidence group independently conducted the literature screening based on the inclusion and exclusion criteria. In the event of any disagreement, the third member of the evidence group would participate and reach a consensus. The inclusion criteria were as follows: (1) clinical practice guidelines and expert consensus; (2) systematic reviews and meta-analyses; (3) original research. The reference criteria were as follows: (1) case series studies and clinical experience summaries; (2) expert opinions published in core Chinese journals and PubMed- and SCI-indexed journals. The exclusion criteria were as follows: (1) non-Chinese/English-language literature; (2) literature that did not involve ultrasound monitoring and the use of fiberoptic bronchoscopy for NFB-BAL; (3) editorials, commentaries, and expert reviews or review lecture research; (4) abstracts of papers, conference papers, papers that interpreted guidelines/consensuses, and letters from readers. A total of 85 articles were finally included. Among them, 5 were related to guideline development methods, and 82 were evidence-based literature supporting the formation of recommendations, including 10 consensus guidelines, 15 systematic reviews/meta-analyses, 51 original studies, and 4 others.

Under the guidance of the experts from the Evidence Evaluation and Methodology Group, the Grading of Recommendations Assessment, Development and Evaluation (GRADE) was used to rate the quality of the evidence and the strength of the recommendations; the experts in the group combined these two aspects to form their recommendations. The quality of the evidence was classified into four levels: high, medium, low, and very low [24,25,26] (Table 1).

**Table 1:** The classification standards for the quality of evidence and recommendation strength adopted in these guidelines [23,24].

Category	Standards
Evidence quality	
High (A)	The actual effect was almost exactly the same as the expected effect.
Moderate (B)	The actual effect may not differ significantly from the expected effect, or there may be a considerable disparity.
Low (C)	The actual outcome may differ significantly from the expected outcome.
Very low (D)	The actual outcome is very likely to differ significantly from the expected outcome.
Recommendation strength	
Strong (1)	The expert team has full confidence in this recommendation. After implementing the intervention measures, the benefits will clearly outweigh the drawbacks.
Weak (2)	The expert team has some confidence in this recommendation. After implementing the intervention measures, the benefits are likely to outweigh the drawbacks, or they may be almost equal.

### 2.8. Eligible Participants and Target Population

These guidelines could be applicable to neonatologists, pediatricians, nurses, and respiratory therapists. The target population was newborn infants who were diagnosed with APDs via LUS in a neonatal ward.

### 2.9. Selection and Final Determination of Clinical Issues

First, the experts from the preparation team, using key technologies involved in the NFB-BAL technique—which have long been studied—in addition to the relevant literature, initially identified related clinical issues. Thirty clinical issues were identified based on the opinions of clinical physicians and nurses, and an additional 20 clinical issues specifically pertaining to this technology were identified during an online meeting among the preparatory group experts. These issues covered all key steps of LUS-guided NFB-BAL. A first-round Delphi questionnaire was designed on the basis of the 20 issues identified, and a second-round investigation was conducted on the basis of the results of the Delphi survey.

### 2.10. Delphi Questionnaire Survey and Final Recommendation Formation

**Figure 1:**
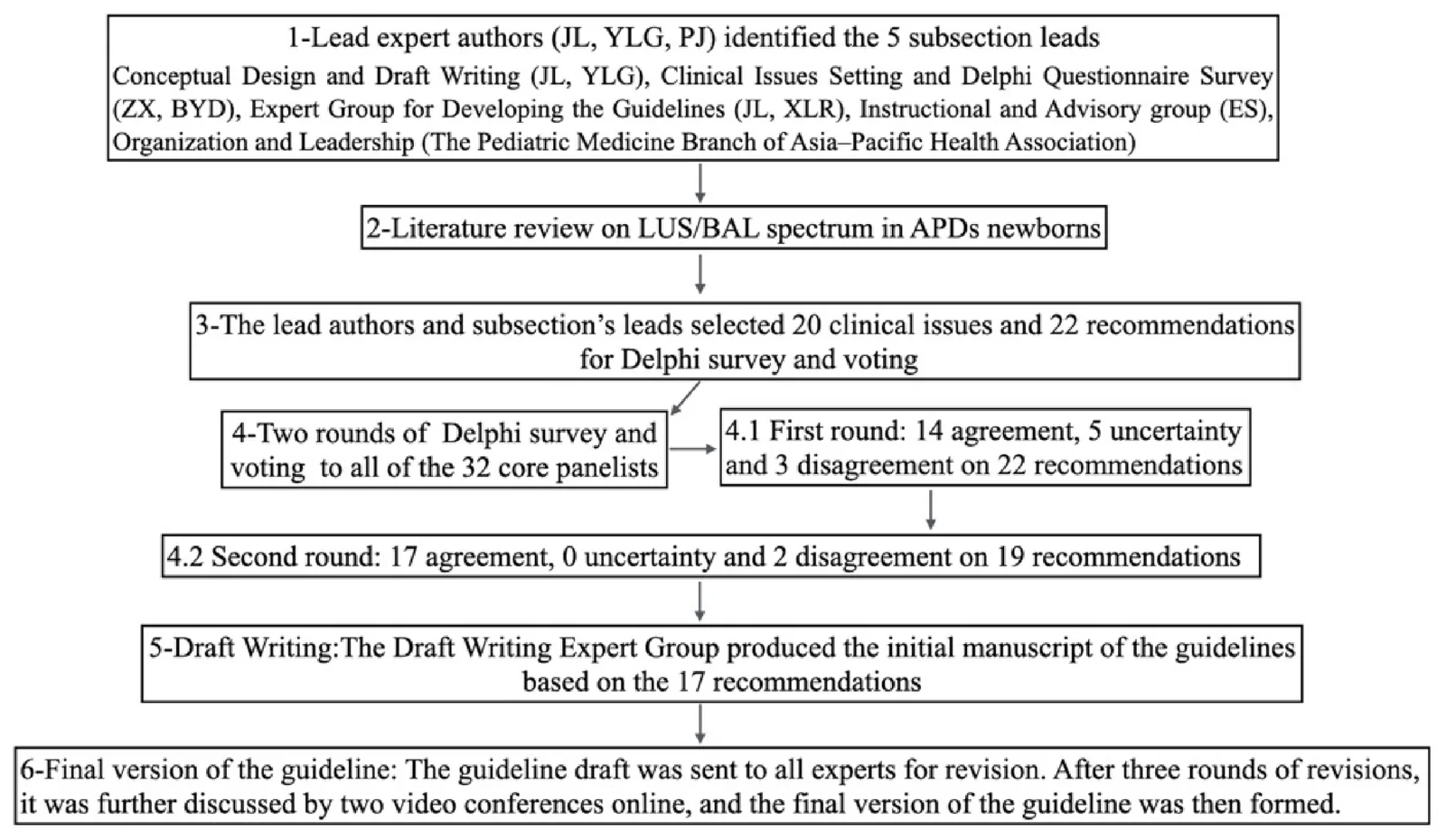
Flow diagram summarizing the steps applied to reach the consensus agreement.

The results of both rounds of the Delphi questionnaire were aggregated, and each expert reported their degree of agreement as follows: agreement, uncertainty, and disagreement. The proposed percentage of expert consensus (%) was calculated as follows: number of experts in agreement/total number of experts × 100%; opinions marked as “uncertainty” or “disagreement” were not included in the consensus. Generally, a proposed percentage of expert consensus greater than 70% is considered sufficient. However, to enhance the representativeness and authority of consensus, the proposed percentage should be greater than 85%. If the threshold was not reached, the statements would be revised based on the experts’ feedback and resubmitted for a second round. Statements that did not reach the predefined consensus threshold after the third round were classified as “no consensus”. Finally, 17 recommendations were formed for 13 key clinical issues. The recommendation strength grading criteria referred to in this consensus are shown in Table 1 [24,25,26], and the flow diagram summarizing the steps applied to reach the consensus agreement is listed in Figure 1.

### 2.11. Obtaining, Disseminating, and Updating the Guidelines

These guidelines will be published in the form of freely accessible. They will also be disseminated at various academic conferences, in training courses, online, and through multimedia channels. The problems encountered during the application of the guidelines and their impact on decision-making will be identified, and on the basis of new evidence, an update will be made approximately every 5 years.

### 2.12. Statement on Funding Support and Conflicts of Interest

These guidelines were developed without any financial support at any stage of their creation, and all experts signed written statements confirming that they had no conflicts of interest.

### 2.13. Limitations and Suggestions for Future Study

The NFB-BAL procedure itself is highly dependent on the operator’s technical proficiency and experience. In the future, high-quality prospective multi-center large sample randomized controlled studies and cohort studies, as well as other types of research, must be conducted to improve the quality and extent of existing evidence; at the same time, various forms of training should be required to overcome technical barriers and to popularize and promote this technology. Second, these guidelines are applicable only to hospitals where the application of LUS is well-established and where BAL technology is available. Resource-limited settings, as well as hospitals that still rely solely on chest X-rays (CXRs) for diagnosing lung diseases and currently do not use LUS in clinical practice, can be used as a reference.

## 3. Clinical Issues and Recommendations

**Question 1:** To diagnose neonatal APDs, should LUS or chest X-ray examination be the preferred method?

**Recommendation 1.** LUS is recommended as the preferred imaging examination method for neonatal APDs (1A: unanimous agreement among experts (100%)).

**Rationale for recommendation**: Increasing evidence indicates that the accuracy and reliability of LUS for diagnosing various lung diseases are superior to those of CXR in all age groups, including newborns, children, and adults [27,28,29,30,31,32,33,34,35,36,37]. A large-scale systematic review and meta-analysis involving 4436 individuals (including newborns, infants, and children), including prospective studies, randomized controlled trials, and multi-center studies, revealed that the sensitivity and specificity of LUS in pneumonia diagnosis were 95% (95% CI = 92–97%) and 94% (95% CI = 88–97%), respectively [31]. Both the specificity and sensitivity were higher than those of CXR (92% vs. 93%) [32]. With respect to pulmonary atelectasis, the study showed that LUS could detect 100% of cases, whereas the sensitivity of CXR examination was only 75% [35]. When diagnosing other lung conditions such as pneumothorax, LUS also demonstrated higher sensitivity and specificity compared to CXR; a systematic review and meta-analysis showed that the overall specificity and sensitivity of lung ultrasound in the diagnosis of neonatal pneumothorax was 98% (95% CI = 0.94–0.99) and 99% (95% CI: 0.98–1.00), respectively. The diagnostic odds ratio was 920.01 (95% CI: 265.81–3184.33), and the area under the curve was 0.996 7 (Q = 0.978 5). However, CXR was always taken as the reference standard with a sensitivity of 82% (95% CI = 0.72–0.90), a specificity of 96% (95% CI = 0.90–0.99), and a diagnostic odds ratio of 44.54 (95% CI: 4.30–460.98). Overall, compared with CXR, LUS has more significant advantages in the diagnosis, differential diagnosis, and guidance of treatment and nursing care for neonatal lung diseases; it is shaping clinical decision-making and redefining best practices in pulmonary care by minimizing invasive procedures and reducing radiation exposure [38,39,40]. Using LUS instead of X-ray to diagnose neonatal lung disease was believed to be an important milestone in the development of neonatal medicine and it should be established as the gold standard for the diagnosis of respiratory diseases in neonates [41,42]. For the methods used for lung ultrasound examination, equipment selection, parameter adjustment techniques, and diagnostic methods and standards for lung diseases, readers can refer to the published guidelines [36,37,43,44,45,46,47,48].

**Question 2**: What Are the Indications and Contraindications for NFB-BAL in Neonatal APDs?

**Recommendation 2:** When indicated, NFB-BAL should be performed under LUS guidance in infants with APD (1A: unanimous agreement among experts (100%)).

**Recommendation 3.** NFB-BAL under LUS guidance should be considered for the following clinical indications (2B: unanimous agreement among experts (92.1–100%)): (1) the patient requires or has already received invasive mechanical ventilation support upon admission; (2) both the patient’s lungs show diffuse consolidation despite non-invasive ventilation maintaining normal blood oxygen saturation; (3) it is difficult to wean the patient off ventilator support, or the patient requires invasive ventilator support again after weaning; and (4) the patient’s arterial blood gas analysis reveals severe hypercapnia (PaCO_2_ > 70 mmHg).

**Recommendation 4.** Experts agree that the following are contraindications or relative contraindications for the NFB-BAL procedure under LUS monitoring: (1) the patient has associated conditions not addressed by these guidelines, such as respiratory distress syndrome (RDS), pulmonary edema, pneumothorax, pulmonary hemorrhage, or congenital bronchopulmonary dysplasia; (2) the patient’s condition is extremely critical, and they cannot tolerate ventilator support interruption for a short period or the cessation of oxygen therapy; (3) severe intracranial hemorrhage at the acute stage; and (4) clinical assessment reveals that the patient’s condition is too critical and unstable for them to undergo the NFB-BAL procedure (1C: unanimous agreement among experts (97.4–100%)).

**Rationale for recommendations**: Multiple prospective case–control studies and retrospective case summaries have revealed that NFB-BAL under LUS monitoring has significant advantages over other methods [2,17,18,19,20,21]. NB-BAL under LUS monitoring offers significant benefits for patients, such as a 56% reduction in invasive ventilation rate, with pooled effect sizes OR = 0.45 (95% CI: 0.22–0.94, *p* < 0.01), I^2^ = 0.0%, *p* = 0.891. Those who still needed to use mechanical ventilation experienced a significantly shorter duration of invasive ventilation (*p* < 0.001). The incidence of severe complications decreased by 75%, with pooled effect sizes OR = 0.25 (95% CI: 0.10–0.59, *p* < 0.01), I^2^ = 10.9%, *p* = 0.287. Patients’ length of hospital stay (LHS) was significantly shortened by 4.5 days, with pooled effect sizes LHS = 4.49 days (95% CI: 5.07–3.91, *p* < 0.001), I^2^ = 0.0%, *p* = 0.533. Meanwhile, hospitalization costs were also reduced accordingly (*p* < 0.001) [20,21]. NFB-BAL technology can significantly reduce the probability of or prevent repeated intubation, lower the incidence of bronchopulmonary dysplasia in premature infants, reduce the need for extracorporeal membrane oxygenation, and decrease the risk of mortality [2,19,20,21,49,50,51,52,53,54]. Clinical results have revealed that for patients with the above-mentioned indications, after receiving NFB-BAL, severe pulmonary consolidation or atelectasis immediately disappeared or significantly improved, and either invasive ventilator support was no longer needed or the duration of support was shortened [2,19,20,21,55]. Avoiding or delaying NFB-BAL often leads to prolonged illness or a poor prognosis and is among the most important causes of complications such as persistent pulmonary hypertension in newborns and iatrogenic bronchopulmonary dysplasia in premature infants [55,56]. According to recently released guidelines, NFB-BAL procedures should be performed to reduce the use of ventilators and other related complications in newborn infants with indications [56].

The NFB-BAL procedure shows remarkable efficacy in treating APDs, but it is not suitable for RDS (herein, RDS refers to pulmonary hyaline membrane disease caused by pulmonary surfactant deficiency), pulmonary edema, pneumothorax, or pulmonary hemorrhage [2,19,20,21,49,50,51,52,53,54]. Owing to the pathogenesis of and pathological changes associated with RDS, lavage may aggravate these diseases; they also do not fall within the scope of APDs, as defined by consensus in this study. Although no cases of intracranial hemorrhage or worsening hemorrhage were observed during long-term clinical practice, it remains necessary to protect the infant’s head and neck during the lavage process, as hemodynamic instability is closely related to intracranial hemorrhage in premature infants [57,58]. Because premature infants are of younger gestational age and lower birth weight, the subependymal matrix in these infants is less developed. Therefore, measures to stabilize and reduce stimulation should be implemented first. Hence, for infants at the acute stage of severe intracranial hemorrhage, NFB-BAL should be postponed.

**Question 3:** How Should One Choose the NFB-BAL Solution?

**Recommendation 5.** Experts recommend using a sterile irrigation solution of 0.9% NaCl at 37 °C for NFB-BAL (1A: unanimous agreement among experts (92.1%)).

**Recommendation 6.** An exogenous pulmonary surfactant (PS) diluent is recommended as the lavage fluid for any of the following indications (1B: unanimous agreement among experts (100%)): (1) an unsatisfactory effect of 0.9% NaCl irrigation after two to three courses; (2) a prolonged disease course, and severe lung consolidation with a large consolidation area; and (3) prematurity with an extremely low birth weight.

**Recommendation 7.** When a PS diluent is used as the lavage fluid, a PS concentration between 12 and 20 mg/mL is recommended (2C: unanimous agreement among experts (100%)).

**Rationale for recommendations:** Usually, the use of sterile 0.9% NaCl for irrigation can achieve good results for most early cases [2,18,19,20,21]. A total of 437 patients who underwent NFB-BAL using 0.9% NaCl as the lavage fluid were included in this study. The lavage efficacy was evaluated on the basis of the categories of marked improvement, improvement, and no improvement. The results revealed that 437 patients achieved a marked improvement or improvement, with a total effectiveness rate of 95% [2,18,19,20,21]. Even for lavage procedures conducted for specific purposes (e.g., biochemical testing, pathogenic microbe detection, toxicology testing, and cytological morphological examination), the use of neutral and isotonic buffers with a pH of 7.4, such as 0.9% NaCl or phosphate-buffered solution [2,18,19,20,21,59,60], is recommended. Therefore, these guidelines recommend 0.9% NaCl as the preferred lavage solution, and maintaining the temperature of the lavage solution at 37 °C is advised [56].

*For infants with late-stage refractory conditions, a diluted PS solution can be used for lavage.* Although RDS is an absolute indication for the use of exogenous PS, various types of lung injury can lead to secondary PS deficiency. Therefore, for severe MAS, severe pneumonia, and atelectasis, PS supplementation therapy can be applied [18,19,20,21,49,50,51,52,53,61,62]. Early animal experiments have already revealed that lavage with a diluted surfactant solution effectively washed out meconium, improved gas exchanges, and improved the histological and radiological appearances in a rabbit model of MAS [63]. Clinical studies have shown that exogenous PS has significant advantages in terms of shortening the duration of ventilator support for patients with these diseases, reducing complications, and shortening the length of hospital stay [49,50,51,52,61,62]. Therefore, for patients who need a PS diluent solution, if no satisfactory effect is achieved after one course of irrigation (applied consecutively two to three times), one more dose of PS preparation can be added after each course of irrigation. During the PS treatment period, conventional or high-frequency invasive ventilation can be used according to the condition of the infant.

*The concentration of PS should be appropriate*. There is no evidence suggesting that NFB-BAL itself can result in PS dilution or deficiency. However, severe pulmonary lesions can affect the synthesis and secretion of PS. Moreover, very-low-birth-weight premature infants may have PS deficiency. Therefore, for severe pulmonary consolidation (with the consolidation area exceeding 50% of the lung field and the air bronchogram disappearing within the consolidation area) or for infants who do not respond well to 0.9% NaCl lavage, PS lavage may be a better option [49,50,51,52,53]. It can reduce the time required for lung re-expansion, the duration of ventilator support and oxygen therapy, the incidence of complications (such as pneumothorax, pulmonary hemorrhage, and persistent fetal circulation) and mortality, and the need for extracorporeal membrane oxygenation [49,50,51,52,53]. There are various reports on the dosage or concentration of PS used during bronchoalveolar lavage. Some case–control studies or systematic review analyses have shown that while the use of a PS concentration of 5 mg/mL is effective, it may not achieve the expected results [64]. In another case–control study on the use of diluted PS lavage for treating MAS, the researchers used a PS concentration of 12 mg/mL and reported that an ideal effect could be achieved with this approach [52]. Another study revealed better outcomes with a dosage of PS of 50 mg/kg per application [53]. However, the dose of PS used in this study was significantly greater, reaching the therapeutic dose for RDS, as reported in the literature when monitored via ultrasound [65]. On the basis of the above analysis, to fully utilize the advantages of the PS dilution solution for irrigation while avoiding waste or excessive use of PS, the use of a PS dilution concentration of 12–20 mg/mL is recommended.

**Question 4**: How Should One Determine the Lavage Fluid Dosage for NFB-BAL?

**Recommendation 8.** A lavage solution dosage of 1.0–2.0 mL per kilogram per lavage is recommended (1C: unanimous agreement among experts (100%)).

**Rationale for recommendation**: Multiple prospective case–control studies and retrospective experience summaries including 437 neonatal patients have shown that if diagnosis and treatment are timely, 0.9% NaCl at a dosage of 1.0–2.0 mL/kg per time can achieve an ideal effect [2,18,19,20,21]. Individuals with a lower body weight can usually start with a higher dose, whereas those with a higher body weight can start with a lower dose. However, in the event that a PS dilution solution is used for lavage in the treatment of MAS, the dosage of lavage fluid can reach 20–30 mL/kg per time [50,53]; owing to the large amount of fluid that enters the lungs in a short period, the child will undoubtedly experience ventilation and gas exchange disorders, as well as decreased blood oxygen saturation and a decreased heart rate [49,51,53]. Furthermore, although these studies used a larger volume of lavage fluid, they used a smaller dose of PS (5 mg/kg each time), thus resulting in limited therapeutic effects. Experimental studies involving animals (rabbits with a weight comparable to that of a newborn) have shown that when 0.9% NaCl is injected into the lungs at a dose of 4 mL/kg, LUS may show B lines. When the dose reaches 6 mL/kg, the breathing rate increases; when it exceeds 8 mL/kg, type I respiratory failure occurs; when it exceeds 10 mL/kg, the heart rate decreases and type II respiratory failure occurs; when it exceeds 15 mL/kg, the rabbit’s breathing stops; and when it exceeds 20 mL/kg, the rabbit’s heart stops beating [66,67]. Therefore, according to the above literature and expert opinions from the Delphi questionnaire survey and to ensure the clinical stability of and reduce the risk of hypoxemia and hemodynamic instability in neonates, particularly in extremely preterm infants, a dosage of 1.0–2.0 mL/kg should be administered regardless of whether 0.9% NaCl or PS diluent is used as lavage fluid.

**Question 5:** What Is the Specific Process for NFB-BAL?

**Recommendation 9.** The process for NFB-BAL under LUS monitoring is as follows (1B: unanimous agreement among experts (100%)): (1) Place the infant in the appropriate position on the basis of LUS findings. (2) Initiate electrocardiogram to ensure the safety of the infant during the procedure. (3) Before injecting the lavage fluid, adjust the ventilator parameters in those currently receiving ventilator support, and re-intubate those who have not received or are currently weaned from ventilator support for invasive positive pressure ventilation. (4) Inject the lavage fluid into the tracheal tube, continue positive pressure ventilation for 20–30 min, then perform continuous rapid percussion on the chest where the lung lesion is located, and then conduct endotracheal suctioning under negative pressure. Each complete cycle is regarded as one course of lavage. (5) After each lavage, repeat LUS immediately. Lavage can be stopped if lung consolidation has almost disappeared; otherwise, another lavage should be performed. Continuous lavage should not be performed more than three times, which is regarded as one treatment course. (6) On the basis of the recovery status of lung consolidation, a new course of lavage can be repeated every 6 to 12 h, and invasive conventional or high-frequency mechanical ventilation should be continued between the two courses.

**Rationale for recommendation:** (1) The patient’s position can affect the outcome of lavage. If both the left lung and the right lung have obvious lesions, then the infant can be placed in a supine position with the midline; if the lesion is mainly in one lung, then the infant can be placed in a lateral position with the affected side facing down. If the lesion is located mainly in the lower lung field, the infant can be placed in a head-high and foot-low position; conversely, if the upper lung field of the infant is more severely affected, then the infant can be placed in a head-low and foot-high position. Lavage should be performed on an empty stomach or between two feedings to prevent gastric reflux, vomiting, and aspiration during the back-tapping and suctioning process. If necessary, the gastric contents can be aspirated before lavage.

(2) Conventional frequency mechanical ventilation should be employed, and the ventilator parameters should be appropriately increased during the lavage process. The purpose of using conventional positive pressure ventilation during lavage is to ensure that the lavage fluid injected into the endotracheal tube can fully enter the hyperinflated and non-contracted lung tissue. Therefore, during NFB-BAL, the ventilator parameters need to be appropriately increased. In general, on the basis of the weight of the patient, the following criteria are applied: an inspiratory peak pressure ≥ 25–28 cmH_2_O; an expiratory end positive pressure ≥ 5–6 cmH_2_O; a respiratory rate ≥ 40 times/min; and an inspiratory time ≥ 0.50 s. The oxygen concentration for inhalation should be adjusted according to the patient’s degree of oxygen dependence [2,18,19,20,21,55]. If the peak inspiratory pressure (PIP) is too low for the lavage fluid to enter the consolidated lung tissue, the desired lavage effect cannot be achieved. In long-term clinical practice, no complications, such as air leakage caused by an increased PIP, have been revealed [2,18,19,20,21]. Lung re-expansion strategies that have been frequently adopted in recent years, including for premature infants with a gestational age of 23–24 weeks, also require a relatively high average airway pressure to achieve the expected results [64,68]. The duration of positive pressure ventilation after lavage fluid injection into the tracheal tube can start at 20 min, on the basis of the degree of lung consolidation and atelectasis. If the initial lavage effect is not satisfactory, it can be extended to 30 min. If necessary, additional lavage can be performed during the positive pressure ventilation period.

(3) Precautions for chest percussion: ① It is usually necessary to temporarily disconnect the ventilator when performing chest percussion for the following reasons. The positive pressure of the ventilator makes it difficult for sputum and secretions from the trachea and bronchi to flow out, thereby exacerbating the ventilation disorder; ② during the back-pumping process, air leakage may occur because of resistance from the ventilator or cough reflexes; and ③ the unstable tip of the endotracheal tube may cause damage to the tracheal mucosa. When the patient’s overall condition is poor, chest percussion can be performed while the patient is connected to the ventilator. In this case, the procedure should be performed carefully. The chest percussion process should be performed by two people working together. One person taps the affected area and subsequently performs negative pressure suction, while the other person stabilizes the patient’s head, neck, tracheal tube, and buttocks to provide stability and protection.

(4) The methods and techniques of chest percussion are very important. The location of the lung lesions, as observed during LUS examination, is the point used for percussion. This is not the traditional method of percussion, in which the entire chest is percussed from bottom to top and from outside to inside. An appropriate non-inflatable infant mask should be used for chest percussion. The strength of the wrist should be used to drive the strength of the arm so that sufficient force can be transmitted to the deep part of the chest cavity to vibrate consolidated and non-contracted lung tissue. The frequency of chest percussion should be no less than 100–120 times per minute. During the tapping process, the spine should be avoided. The chest percussion force should be moderate. If the force is insufficient, the deep parenchymal lung tissue will not vibrate, and if the force is excessive, there is a risk of rib fracture, especially in low-gestational-age premature infants and low-birth-weight infants. In such cases, CXR or ultrasound examination can be performed to assist in diagnosis or rule out the condition [69]. Mild pulmonary edema may be a relatively common side effect of NFB-BAL and is related to the severity of the underlying disease and the frequency of lavage. However, the degree of edema is generally not severe, and no special treatment is needed.

(5) Patient safety should be maintained during the suctioning process. The suctioning process should be completed within 10 s and should not exceed 15 s to avoid asphyxia and hypoxia. During sputum aspiration, the negative pressure should be maintained between 80 and 100 mmHg (1 mmHg = 0.133 kPa). The technique should be gentle to avoid damaging the trachea–bronchial mucosa and the suction tube should not be inserted too deep. Rotation, suction, and withdrawal should be performed simultaneously, avoiding up-and-down puncturing. The negative pressure suction tube should disconnect in the event of excessive resistance upon tube withdrawal. In addition to the possibility of the suction tube being blocked because of the viscosity of the sputum plug, in a few cases, blockage may be caused by excessively high negative pressure, resulting in blockage of the side hole of the suction tube due to compression of the airway–bronchial mucosa under negative pressure suction. If suction is continued, mucosal damage can occur. Furthermore, the operator’s skills and proficiency can significantly influence the effectiveness of the lavage process, including the adjustment of ventilator parameters and the force, amplitude, and frequency of chest percussion. Therefore, before NFB-BAL is performed, training is necessary, as this is key to achieving good results.

(6) The appropriate number of courses of lavage should be considered. If the effect of one course of lavage is not satisfactory (not reaching the criteria for weaning from the ventilator), multiple courses of lavage may be needed, and invasive ventilator support should continue [18,19,20,21]. After one course of treatment, the parameters of the ventilator can be appropriately adjusted, and an equal dose of lavage fluid can be injected. Afterward, with constant positive pressure ventilation for 20–30 min, the ventilation mode can be changed to high-frequency oscillation ventilation until the next course of lavage (that is, high-frequency ventilation is given between two courses). Depending on the condition, one course can be repeated every 6–12 h (that is, two to four courses per day) [2,18,19,20,21]. If two to three lavage procedures are performed within one treatment course, arterial blood gas analysis should be repeated after the lavage process has been completed. However, high-frequency ventilation can be temporarily applied if the PaCO_2_ is <30 (especially <26) mmHg to avoid a significant decrease in the cerebral blood flow of patients. For patients with severe lung consolidation and poor lavage results who require multiple lavage treatment courses, appropriate exogenous PS preparations can be supplemented after lavage to promote lung re-expansion. The dosage should be 50% of the traditional dose [56,65].

**Question 6:** During NFB-BAL, what issues can arise that would require the operation to be paused?

**Recommendation 10.** NFB-BAL should be immediately stopped if any of the following situations occur (2B: unanimous agreement among experts (97.4–100%): (1) the infant presents obvious cyanosis; (2) percutaneous blood oxygen saturation monitoring indicates a decrease in blood oxygen saturation of more than 10% or a saturation level below 80%; and (3) electrocardiogram monitoring reveals a heart rate of less than 90 beats per minute.

**Rationale for recommendation**: NFB-BAL must be performed in a manner that ensures the safety of the patient. Any situation that may lead to the aggravation or deterioration of the patient’s condition must be avoided. The procedure must be paused if the patient shows obvious cyanosis, a significant decrease in blood oxygen saturation, or a marked decrease in heart rate during the NFB-BAL procedure [17,51,52]. In this situation, the ventilator should be connected, and LUS (combined with cardiac ultrasound if necessary) should be promptly performed to identify the cause and to take appropriate measures. Once the patient’s condition has stabilized, treatment can continue.

**Question 7:** How can pulmonary atelectasis caused by increased mucus or secretions in the respiratory tract be managed?

**Recommendation 11.** The following stepwise approach for the management of pulmonary consolidation or atelectasis caused by sputum or secretion blockage is recommended (1C: unanimous agreement among experts (97.4–100%)): (1) First, through direct observation through a laryngoscope, a suction tube should be inserted into the main bronchus. Afterward, the mucus plug and secretions in the trachea should be removed under negative pressure. (2) Second, tracheal intubation should be performed if the effect is not satisfactory, and an aspirating tube should be used to remove the thick and viscous phlegm and secretions from the deep part under negative pressure suction. (3) Finally, the NFB-BAL procedure should be performed promptly if the above measures remain ineffective.

**Rationale for recommendation**: Pulmonary atelectasis caused by retained secretions in neonates is managed initially with gentle airway suctioning, adequate humidification, optimal positioning, and ventilatory support. Chest physiotherapy and nebulized saline may be used selectively. In intubated infants with persistent atelectasis, NFB-BAL using small, weight-based saline volumes can facilitate secretion clearance and diagnostic sampling while minimizing physiological instability. Occasionally, selective intubation should be performed if the effect is not complete, while bronchoscopy is reserved for refractory cases.

Low-birth-weight and small-for-gestational-age premature infants may have weak cough reflexes, poor expectoration capabilities, and gastroesophageal reflux. Additionally, they often receive various forms of non-invasive respiratory support, which makes mucus and secretions thicker and more difficult to clear. This leads to obstruction of the respiratory tract, causing pulmonary consolidation and atelectasis, subsequently resulting in respiratory distress or worsening respiratory difficulties and even the need for reintubation [2,21]. In this situation, it is necessary to promptly repeat LUS. The recommended measures should be immediately implemented, which usually lead to good results if pulmonary consolidation or atelectasis is present [2,21]. If not detected in time, pulmonary consolidation and atelectasis will worsen. Afterward, the NFB-BAL procedure must be performed to facilitate rapid re-expansion of the lungs if this simple treatment is ineffective. After the above-mentioned procedure is complete, no additional respiratory support is needed if the lung lesions have resolved and if the infant is stable. Continuous LUS monitoring post-extubation is important for guiding support decisions.

**Question 8:** What Are the Indications for Weaning from Invasive Ventilation After NFB-BAL?

**Recommendation 12.** When LUS shows that either of the following parameters after lavage has been achieved, the invasive ventilator can be removed (2B: unanimous agreement among experts (97.4%): (1) lung consolidation has completely resolved, or (2) if lung consolidation has not completely disappeared, its extent does not exceed two intercostal spaces, or it involves an area beneath the pleura (subpleural small consolidations) in only one or multiple intercostal spaces.

**Figure 2:**
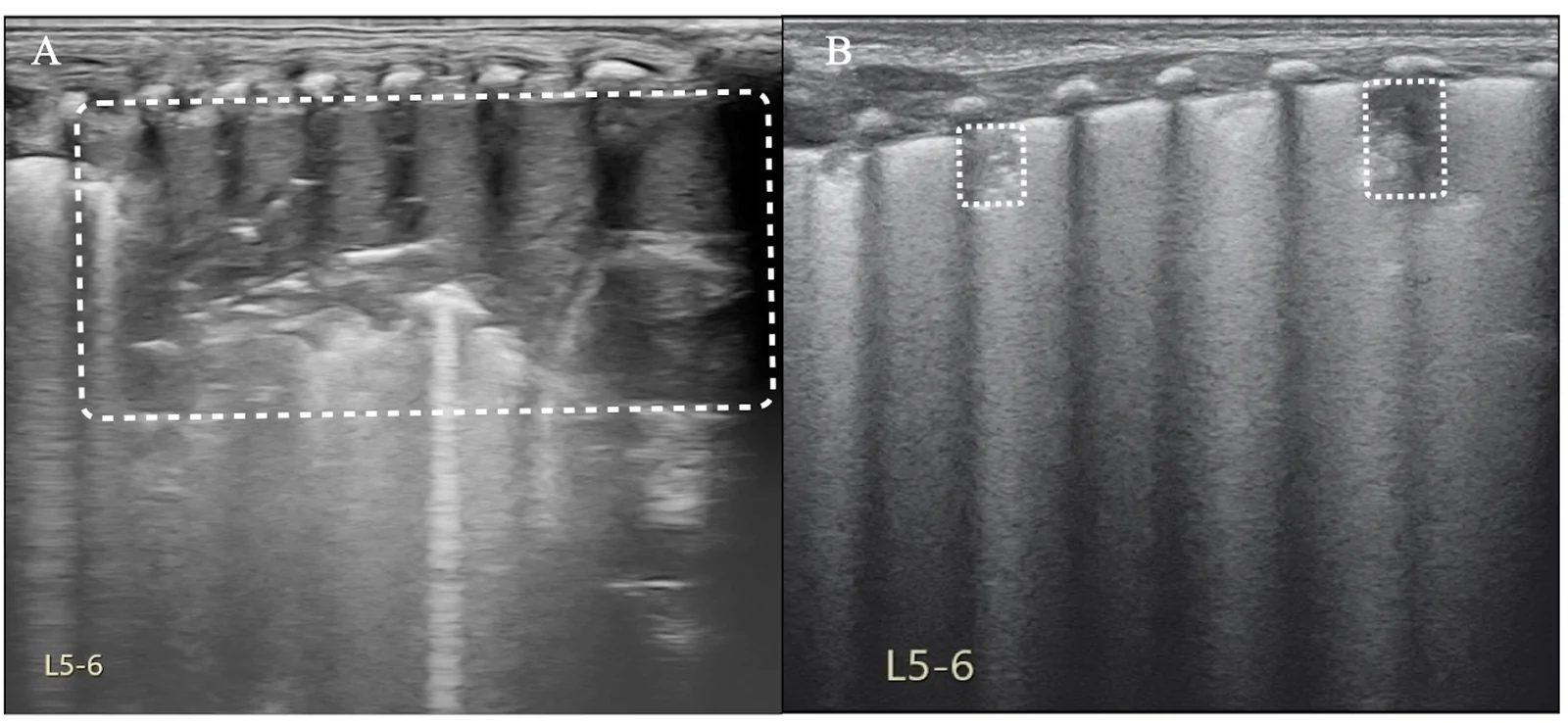
Lung consolidation was significantly reduced but did not completely disappear after NFB-BAL. This figure depicts a premature infant with a gestational age of 26 weeks. The baby was transferred to the NICU under invasive mechanical ventilation because of respiratory distress. LUS examination revealed extensive consolidation and atelectasis in her left lung ((**A**), dashed box) at 2 weeks after birth because of ventilator-associated pneumonia. However, only the lung had localized consolidation limited to the pleura after NFB-BAL was administered three times ((**B**), dashed box), allowing the invasive ventilator to be withdrawn.

**Rationale for recommendation**: After each lavage, LUS should be repeated immediately. Whether it is best to remove the invasive ventilator after lavage depends on ultrasound examination results and should be determined on the basis of successful lung re-expansion rather than following traditional weaning criteria [1,2,70] (Figure 2), as traditional indications for weaning and current weaning methods are not suitable for cases in which lung diseases are managed under the guidance of ultrasound monitoring [1,2,54,62]. In clinical practice, successful lung re-expansion is usually defined as complete re-expansion, meaning that during LUS, lung consolidation or atelectasis has completely disappeared, and the ultrasound image is almost normal; partial re-expansion refers to the presence of a large number of bronchial air-trapping signs, a significant increase in bronchial air-trapping signs in the original lung consolidation area, or a significant reduction in the lung consolidation area compared to beforehand, with a few A-lines or a fusion of B-lines being the main manifestation within the consolidation area. LUS images can show whether there are any lesions in the lungs and the severity of those lesions so that medical experts can then decide whether to stop ventilatory support, which in turn can reduce the need for further ventilatory support, shorten the time of intubation, and avoid repeated intubation [1,2,18,19,20,21,54]. This is also one of the reasons why it is recommended that LUS be repeated after each course of irrigation.

**Question 9:** Is it necessary to provide a child with non-invasive respiratory support after removal from invasive ventilation support? What are the indications for this?

**Recommendation 13.** Non-invasive respiratory support should be considered after removal from invasive ventilation support under the following circumstances (1C: unanimous agreement among experts (97.4%)): (1) lung consolidation has not completely resolved, and there is still significant breathing difficulty even after removal from invasive ventilation support; (2) the patient has multiple organ system disorders that require respiratory support; and (3) for small premature infants and low-birth-weight infants with thoracic underdevelopment high compliance, external positive support is needed to enhance thorax stability.

**Rationale for recommendation**: Traditionally, after patients are weaned from invasive ventilators, clinicians often provide non-invasive respiratory support to prevent recurrent breathing difficulties. In fact, for patients without pulmonary lesions, unless their thoracic stability is extremely poor, no additional respiratory support is usually needed. Moreover, LUS reveals the recovery status of pulmonary lesions in newborn infants [1,2,18,19,20,21]. After removal from an invasive ventilator, continuous monitoring of the changes in lung ultrasound is still necessary. In particular, for infants who experience breathing difficulties again after being weaned from a ventilator, determining whether such breathing difficulties are caused by the recurrence of lung lesions, particularly due to the obstruction of sputum, is crucial. This is particularly important for low-birth-weight and small-for-gestational-age premature infants.

**Question 10:** What Are the Suggestions for Performing the NFB-BAL Procedure on Extremely Premature Infants?

**Recommendation 14.** For extremely premature infants with a gestational age of less than 24 weeks, NFB-BAL is not recommended unless there is a strong clinical indication and if a careful clinical risk–benefit assessment is performed prior to the procedure (2C: unanimous agreement among experts (97.4%)).

**Rationale for recommendation**: Although there is no evidence suggesting that a gestational age of less than 24 weeks is a contraindication for NFB-BAL, on the basis of the following information, 97.4% of experts involved in the consensus recommendation suggest that for extremely premature infants with a gestational age of less than 24 weeks, it should be avoided in the first week after birth, and if truly needed after the first week after birth, then a careful assessment comparing the advantages and disadvantages on the basis of the LUS results is necessary. Liao et al. studied lavage in premature infants with a gestational age of ≥24 weeks [17] and reported the following results. (1) These newborn infants usually have more severe conditions and a greater dependence on ventilators, making it difficult for them to tolerate the interruption of ventilatory support for even a short period. (2) Various stimuli during the lavage process may cause or aggravate cerebral hemodynamic stability, leading to or exacerbating intracranial hemorrhage. (3) For extremely premature infants with a gestational age of 24 weeks or younger, minimizing the stimuli as much as possible within the first week after birth is crucial for ensuring their stability.

**Question 11**: Does Lavage Require Adherence to Aseptic Principles?

**Recommendation 15.** During the NFB-BAL irrigation process, strict aseptic techniques should be maintained, which includes institutional infection protocols for the equipment and environment (1A: unanimous agreement among experts (100%)).

**Rationale for recommendation**: Neonatal infections, especially those occurring in a hospital setting, need to be closely monitored in neonatal intensive care units for the following reasons. During the irrigation process, all operations should adhere strictly to the principles of asepsis [71,72]. First, lung infections, especially ventilator-associated pneumonia, are the main causes of lung consolidation and atelectasis in newborns. Second, critically ill infants in the intensive care unit are at increased risk for infectious diseases, including nosocomial infections. Therefore, the irrigation process must strictly adhere to aseptic principles. The ultrasonic probe and its connecting cables should be disinfected thoroughly to prevent cross-infection. Where necessary, bedside isolation measures should also be implemented.

**Question 12:** Is It Necessary to Conduct Microbiological Testing on the Lavage Fluid?

**Recommendation 16.** The collection of lavage fluid for microbiological testing is recommended under the following circumstances (1A: unanimous agreement among experts (100%)): (1) severe systemic infection or the presence of high-risk factors for sepsis, and the pathogen cannot be identified by routine examinations; (2) conventional treatment is ineffective, and a special pathogen infection, mixed infection, or drug-resistant bacterial infection is suspected; and (3) immunodeficiency or dysfunction of the immune system is present.

**Rationale for recommendation**: Infectious diseases are the main causes of atelectasis in newborns, and antimicrobial treatment is a fundamental treatment measure for these conditions. It cannot be ruled out that patients may be infected with drug-resistant bacteria or rare pathogenic microorganisms. In particular, children with poor immune function are more prone to infections by various pathogenic microorganisms. Microbiological culture examinations such as microbial culture, PCR testing, and even metagenomic testing can be conducted by collecting aspirated fluid during lavage when necessary [15,16,17,73,74,75,76,,77,78,79,80,81,82]. In one study, BAL fluid was collected from 109 neonates with severe pneumonia and subjected to microbiological testing. The results revealed that 69 cases (63.3%) of neonatal severe pneumonia were caused by different pathogenic microorganisms; among them, G-negative bacilli accounted for 81.2% (including *Escherichia coli*, *Klebsiella pneumoniae*, and *Pseudomonas aeruginosa*), G-positive cocci accounted for 15.9% (mainly *Staphylococcus aureus*), and fungi accounted for 2.9% [75]. A multi-center study conducted in Hong Kong, Beijing, and Canada involved 18 patients with a compromised immune system. A total of 21 lavage fluid microbiological tests were performed on these children. Pathogenic microorganisms were detected in 14 patients (66.7%), while seven patients (33.3%) had negative test results. Among these, 12 cases presented one type of pathogenic microorganism, and two cases presented two types, including seven cases of bacteria, eight cases of viruses, and one case of fungi [76]. Patients who have persistent cough, gastroesophageal reflux, chronic pneumonia, and dysphagia may need to have the lavage fluid collected for cytological or pathogenic examination. In short, microbiological detection in bronchoalveolar lavage fluid is highly important for clarifying the etiology of patients with difficult-to-treat conditions [77,78,79].

**Question 13:** In what circumstances is it necessary to use a fiberoptic bronchoscope for bronchoalveolar lavage under LUS monitoring?

**Recommendation 17.** Bronchoscopic BAL under LUS monitoring is recommended when the following indications are present (1A: unanimous agreement among experts (100%)): (1) After performing three to four courses of irrigation daily using PS dilution for NFB-BAL, if the effect is still unsatisfactory after continuous irrigation for more than 3 days. (2) When the disease course is long, the degree of lung consolidation is extremely severe, and even with high-parameter invasive ventilator support, increasing the air content in the diseased lung tissue is difficult.

**Rationale for recommendation:** Although most of the patients achieved good results after NFB-BAL, among 317 patients, 17 (5.4%) did not achieve the desired outcome; there was no significant change in pulmonary consolidation or atelectasis after the lavage procedure [2,18,19]. The reasons for this may include the following [2]. (1) The disease course was too long, and the disease was not detected in time. By the time of diagnosis, the degree of pulmonary consolidation had become extremely severe, and the consolidated lung tissue had hardened. (2) Premature infants with a low gestational age may have bronchopulmonary dysplasia (BPD) or weak coughing and expectoration functions. Secretions in the respiratory tract are difficult to expel, and blockage by even a small amount of secretion can cause atelectasis. (3) The infection worsens or recurs, making it difficult to clear sputum or secretions. In such a situation, it is necessary to collect secretions from the airway for bacterial testing. (4) Occult gastroesophageal reflux occurs, causing the stomach contents to be repeatedly inhaled, blocking the respiratory tract. (5) The operator’s skills and proficiency are insufficient. For patients who do not respond to PS lavage or whose condition does not improve with treatment, performing bronchoalveolar lavage via fiberoptic bronchoscopy under LUS monitoring is recommended, which may yield better results [83,84,85].

## 4. Discussion

These guidelines, on the basis of evidence from the literature and expert consensus, provide a detailed introduction to the indications, operation methods, and contraindications for NFB-BAL under LUS monitoring. These advantages include but are not limited to reducing the probability and duration of invasive mechanical ventilation; decreasing the incidence of severe complications such as persistent fetal circulation, pneumothorax, and pulmonary hemorrhage; shortening the length of hospital stay; and reducing hospital costs [20,21,54]. NFB-BAL is also highly important for reducing or avoiding repeated intubation, lowering or preventing the incidence of BPD, reducing the use of ECMO, and minimizing mortality [49,50,51,52]. During the lavage process, the ventilator and oxygen supply need to be temporarily disconnected. Apart from occasional cases in which some newborn patients experienced transient cyanosis, decreased blood oxygen levels, a slowed heart rate, and pulmonary edema, no other side effects or complications related to lavage were observed. Although these side effects usually disappear quickly after the irrigation procedure is stopped and the patient is connected to the ventilator, strict monitoring is still needed. A summary of the clinical issues and recommendations is listed in Table 2.

**Table 2:** Summary of the clinical issues and recommendations.

ClinicalIssues	Recommendations	Evidence Level and Recommendation Strength	Unanimous Agreement Among Experts
To diagnose neonatal APDs, should LUS or chest X-ray examination be the preferred method?	1. LUS is recommended as the preferred imaging examination method for neonatal APDs.	1A	100%
What are the indications and contraindications for NFB-BAL in neonatal APDs?	2. When indicated, NFB-BAL should be performed under LUS guidance in infants with APD.	1A	100%
	3. NFB-BAL under LUS guidance should be considered for the following clinical indications: (1) the patient requires or has already received invasive mechanical ventilation support upon admission; (2) both the patient’s lungs show diffuse consolidation despite non-invasive ventilation maintaining normal blood oxygen saturation; (3) the patient is difficult to wean from ventilator support, or the patient requires invasive ventilator support again after weaning; and (4) the patient’s arterial blood gas analysis reveals severe hypercapnia (PaCO_2_ > 70 mmHg).	2B	92.1–100%
	4. Experts agree that the following are contraindications or relative contraindications for the NFB-BAL procedure under LUS monitoring: (1) the patient has associated conditions not addressed by these guidelines, such as respiratory distress syndrome (RDS), pulmonary edema, pneumothorax, pulmonary hemorrhage, or congenital bronchopulmonary dysplasia; (2) the patient’s condition is extremely critical, and they cannot tolerate ventilator support interruption for a short period or the cessation of oxygen therapy; (3) severe intracranial hemorrhage at the acute stage; and (4) clinical assessment reveals that the patient’s condition is too critical and unstable for them to undergo the NFB-BAL procedure.	1C	97.4–100%
How should one choose the NFB-BAL solution?	5. Experts recommend using a sterile irrigation solution of 0.9% NaCl at 37 °C for NFB-BAL.	1A	92.1%
	6. An exogenous pulmonary surfactant (PS) diluent is recommended as the lavage fluid for any of the following indications: (1) an unsatisfactory effect of 0.9% NaCl irrigation after two to three courses; (2) a prolonged disease course, and severe lung consolidation with a large consolidation area; and (3) prematurity with an extremely low birth weight.	1B	100%
	7. When a PS diluent is used as the lavage fluid, a PS concentration between 12 and 20 mg/mL is recommended.	2C	100%
How should one determine the lavage fluid dosage for NFB-BAL?	8. A lavage solution dosage of 1.0–2.0 mL per kilogram per lavage is recommended.	1C	100%
What is the specific process for NFB-BAL?	9. The process for NFB-BAL under LUS monitoring is as follows: (1) Place the infant in the appropriate position on the basis of LUS findings. (2) Initiate electrocardiogram to ensure the safety of the infant during the procedure. (3) Before injecting the lavage fluid, adjust the ventilator parameters in those currently receiving ventilator support, and re-intubate those who have not received or are currently weaned from ventilator support for invasive positive pressure ventilation. (4) Inject the lavage fluid into the tracheal tube, continue positive pressure ventilation for 20–30 min, then perform continuous rapid percussion on the chest where the lung lesion is located, and then conduct endotracheal suctioning under negative pressure. Each complete cycle is regarded as one course of lavage. (5) After each lavage, repeat LUS immediately. Lavage can be stopped if lung consolidation has almost disappeared; otherwise, another lavage should be performed. Continuous lavage should not be performed more than three times, which is regarded as one treatment course. (6) On the basis of the recovery status of lung consolidation, a new course of lavage can be repeated every 6 to 12 h, and invasive conventional or high-frequency mechanical ventilation should be continued between the two courses.	1B	100%
During NFB-BAL, what issues can arise that would require the operation to be paused?	10. NFB-BAL should be immediately stopped if any of the following situations occur: (1) the infant presents obvious cyanosis; (2) percutaneous blood oxygen saturation monitoring indicates a decrease in blood oxygen saturation of more than 10% or a saturation level below 80%; and (3) electrocardiogram monitoring reveals a heart rate of less than 90 beats per minute.	2B	97.4–100%
How can pulmonary atelectasis caused by increased mucus or secretions in the respiratory tract be managed?	11. It is recommended that the following stepwise approach is used for managing pulmonary consolidation or atelectasis caused by sputum or secretion blockage. (1) First, through direct observation through a laryngoscope, a suction tube should be inserted into the main bronchus. Afterward, the mucus plug and secretions in the trachea should be removed under negative pressure. (2) Second, tracheal intubation should be performed if the effect is not satisfactory, and an aspirating tube should be used to remove the thick and viscous phlegm and secretions from the deep part under negative pressure suction, and (3) finally, the NFB-BAL procedure should be performed promptly if the above measures remain ineffective.	1C	97.4–100%
What are the indications for weaning from invasive ventilation after NFB-BAL?	12. When LUS shows that either of the following parameters after lavage has been achieved, the invasive ventilator can be removed: (1) lung consolidation has completely resolved, or (2) if lung consolidation has not completely disappeared, its extent does not exceed two intercostal spaces, or it involves an area beneath the pleura (subpleural small consolidations) in only one or multiple intercostal spaces.	2B	97.4%
Is it necessary to provide a child with non-invasive respiratory support after removal from invasive ventilation support? What are the indications for this?	13. Non-invasive respiratory support should be considered after removal from invasive ventilation support under the following circumstances: (1) lung consolidation has not completely resolved, and there is still significant breathing difficulty even after removal from invasive ventilation support; (2) the patient has multiple organ system disorders that require respiratory support; and (3) for small premature infants and low-birth-weight infants with thoracic underdevelopment high compliance, external positive support is needed to enhance thorax stability.	1C	97.4%
What are the suggestions for performing the NFB-BAL procedure on extremely premature infants?	14. For extremely premature infants with a gestational age of less than 26 weeks, NFB-BAL is not recommended unless there is a strong clinical indication and if a careful clinical risk–benefit assessment is performed prior to the procedure.	2C	97.4%
Does lavage require adherence to aseptic principles?	15. During the NFB-BAL irrigation process, a strict aseptic technique should be maintained, which includes institutional infection protocols for the equipment and environment.	1A	100%
Is it necessary to conduct microbiological testing on the lavage fluid?	16. The collection of lavage fluid for microbiological testing is recommended under the following circumstances: (1) severe systemic infection or the presence of high-risk factors for sepsis, and the pathogen cannot be identified by routine examinations; (2) conventional treatment is ineffective, and a special pathogen infection, mixed infection, or drug-resistant bacterial infection is suspected; and (3) immunodeficiency or dysfunction of the immune system is present.	1A	100%
In what circumstances is it necessary to use a fiberoptic bronchoscope for bronchoalveolar lavage under LUS monitoring?	17. Bronchoscopic BAL under LUS monitoring is recommended when the following indications are present. (1) After performing three to four courses of irrigation daily using PS dilution for NFB-BAL, if the effect is still unsatisfactory after continuous irrigation for more than 3 days. (2) When the disease course is long, the degree of lung consolidation is extremely severe, and even with high-parameter invasive ventilator support, increasing the air content in the diseased lung tissue is difficult.	1A	100%

It is necessary to reemphasize that APDs are not an independent disease. They refer to a category of diseases characterized by extensive lung consolidation on lung ultrasound imaging, mainly including severe pneumonia, severe MAS, atelectasis, etc. In order to ensure that this technology can be implemented more effectively, a qualification certification and quality improvement system needs to be established. Operators should first receive training in lung ultrasound technology, including training on the basic theory and operational LUS techniques [83,84,85]. Secondly, operators also need to receive training in BAL technology. Only after undergoing relevant technical training and passing the assessment can they officially implement this technology in clinical work. According to our experience, a 3-month training program at a tertiary training center is sufficient to enable a good understanding of neonatal LUS technology, and then roughly 10 cases of BAL operation training are needed to be able to carry out the recommended techniques in this guideline relatively well. In addition, a system for monitoring quality indicators and reporting adverse events should be established and continuously improved, so that this technology can be better applied in clinical practice to maximize the improvement of children’s prognosis.

Importantly, under LUS monitoring, NFB-BAL is not only a therapeutic technique but also a “precise nursing” technique. Therefore, in addition to clinical physicians, key nursing staff in the NICU should understand how to perform both LUS and NFB-BAL. Only when both clinical physicians and key nurses have a strong grasp of these techniques and cooperate with each other in their work can the significant clinical and nursing value be fully demonstrated, thereby preserving the interests of newborns. Both physicians and nurses can perform LUS examinations and are responsible for injecting the lavage fluid into the endotracheal tube. However, the physician is responsible for adjusting the ventilator parameters and protecting the infant during the lavage process, whereas the nurse is responsible for back-tapping and suctioning. Throughout the entire operation, both the physician and the nurse are responsible for observing the infant and the monitoring equipment.

## 5. Conclusions

In conclusion, APDs are common lung diseases in newborns and may lead to serious adverse outcomes. LUS is helpful for their early detection, and implementing NFB-BAL under LUS monitoring can significantly improve their prognosis. In order to enable NFB-BAL technology to be applied in a better and more standardized fashion in clinical practice, experienced experts in this field formulated these guidelines after nearly two years of hard work, which include 17 well-established recommendations. We hope that the wide application of these guidelines will help significantly improve the prognosis of critically ill newborns with atelectatic lung diseases. We also hope that readers will provide feedback. We believe that as this technology is further promoted and applied more widely, there will be more evidence that will be beneficial for future revisions.

## Data Availability

The original contributions presented in this study are included in the article. Further inquiries can be directed to the corresponding author.

## Abbreviations

APDs	Atelectatic pulmonary diseases
MAS	Meconium aspiration syndrome
BAL	Bronchoalveolar lavage
NFB-BAL	Non-fiberobronchoscopic bronchoalveolar lavage
LUS	Lung ultrasound
CXR	Chest X-ray
PIP	Peak inspiratory pressure
BPD	Bronchopulmonary dysplasia
PS	Pulmonary surfactant
